# YouTube and the Achilles Tendon: An Analysis of Internet Information Reliability and Content Quality

**DOI:** 10.7759/cureus.23984

**Published:** 2022-04-09

**Authors:** Kevin M McMahon, Justin Schwartz, Thomas Nilles-Melchert, Kelley Ray, Vincent Eaton, Dennis Chakkalakal

**Affiliations:** 1 Medicine, Creighton University School of Medicine, Omaha, USA; 2 Orthopedics, Creighton University School of Medicine, Omaha, USA

**Keywords:** electronic medical resources, youtube, gqs, jama, tendon rupture, tendonitis, achilles tendon

## Abstract

Purpose: To evaluate the educational content, quality, and reliability of YouTube videos regarding the Achilles tendon and Achilles tendon injuries.

Methods: The first 50 videos found on YouTube after searching “Achilles tendon” were evaluated and classified by content type and uploader source. Reliability and accuracy were assessed using the Journal of the American Medical Association (JAMA) benchmark criteria, nonspecific educational content was assessed via the Global Quality Score (GQS), and specific educational content was assessed using the Achilles Tendon Specific Score (ATSS). ANOVA was performed to determine differences in video reliability and educational content quality by video content type and source. Multivariate stepwise regressions were used to evaluate the effects of specific video characteristics on JAMA benchmark criteria, GQS, and ATSS.

Results: The 50 videos evaluated had a cumulative view total of 53,323,307, a mean of 1,066,466, and a range of 1,009 to 42,663,665 views per video. Most videos focused on disease-specific information (38%) and exercise training (22%). Most videos were uploaded by non-physicians (34%) or medical sources (health websites) (32%). A higher view ratio was an independent predictor of lower JAMA scores (lower reliability and accuracy) (standardized beta = −0.281, P = 0.048) and increased video duration was an independent predictor of greater GQS (standardized beta = 0.380, P = 0.007) and ATSS scores (standardized beta = 0.364, P = 0.009) (increased quality of nonspecific and specific educational content).

Conclusion: Videos on YouTube regarding the Achilles tendon were viewed numerous times, but their educational content and reliability were poor. Providers treating patients for Achilles tendon-related pathologies should initiate a dialogue with patients about their use of internet sources and should educate them on their optimal usage. They should warn them of the low quality of YouTube-derived information and provide them with reliable sources that may better give them control over their own care.

## Introduction

The internet is increasingly prevalent in the lives of Americans with an estimated 85% using it daily, 48% using it multiple times a day, and 31% using it almost constantly as of January 2021 [1]. In a 2013 study, 59% of Americans reported that they looked up healthcare information in the past year with 77% of that group starting with a search engine (e.g. Bing, Google, and Yahoo), 13% starting on medical/specialty sites such as WebMD, 2% on sites such as Wikipedia, and 1% starting on social networks like Facebook [2].

YouTube is one of the most popular sites with 71% of Americans in a study reporting use, and it is being increasingly used by people seeking opinions and facts [3]. With the expansion of the internet and the growth of YouTube as a video service that provides not only entertainment but also news and education, it is becoming increasingly common for people to search out diseases or healthcare-related information on YouTube [4].

Studies have shown that the use of internet sources to obtain healthcare-related information increases with a decreased perceived quality of care and quality of patient-physician relationship [5]. Studies have also reported that only 18% of patients discuss this information with their providers [6]. Use of these internet sources prior to visiting a physician may affect patient expectations of care, presumptive diagnosis, and preferred treatment. This has been evidenced by Perrin and Duggan (2015) stating that 75% of patients with chronic conditions report that their decisions about their treatment were affected by their last online search [7].

Despite the prevalent use of online resources, YouTube has no peer-reviewed process or quality control methods in place for most health-related videos, which may present patients with low quality, misleading, and/or incomplete information [6,8,9]. Previous studies on the educational accuracy of YouTube videos have been conducted on orthopedic topics and conditions such as hip and knee arthritis, articular cartilage defects, lumbar discectomies, kyphosis, and more [6,10-14]. These studies have likewise reported poor educational content in most videos available on YouTube.

This study aims to evaluate the educational content of YouTube videos regarding the Achilles tendon. The Achilles tendon is the largest tendon in the human body, functions in plantar flexion of the foot, and is most commonly injured during athletic activities such as basketball [15]. A recent study by Lemme et al. (2018) reported that Achilles tendon ruptures in the general population increased from 1.8 to 2.5 per 100,000 persons between 2012 and 2016, were most common in males, and had the highest incidence rate in patients aged 20-39 years (5.6/100,000) [15]. Due to the greater incidence of injuries in younger patients and their increased utilization of internet sources over older patients, it is important to evaluate the quality of online videos about the Achilles tendon. The authors of this paper postulated that similar to previous studies on other orthopedic topics, the quality, reliability, and content of YouTube videos on the Achilles tendon would be poor and/or incomplete.

## Materials and methods

The authors of this study searched the term “Achilles Tendon” on YouTube on May 24, 2021. A Google Chrome (Google LLC, Mountain View, CA) incognito tab was used to eliminate any confounding factors influencing the results. The videos were queued by the default filtering method of “relevance” and the first 50 videos were recorded for evaluation (Appendix). This is a reasonable approach as previous studies on orthopedic-related topics have been similarly conducted and accepted in peer-reviewed journals [6]. Exclusion criteria included any video in a non-English language or videos consisting only of audio content. If these criteria occurred, the next acceptable video was recorded and evaluated.

Video characteristics

Video characteristics were recorded for each of the following variables: title, video duration (in minutes), number of views, video source, content type, days since upload, view ratio (views per day), number of likes, number of dislikes, like ratio ((likes * 100) ÷ (likes + dislikes)), and video power index (VPI). The VPI uses the following formula: like ratio * view ratio ÷ 100; it has been utilized in prior studies to measure video popularity by views and likes [6].

Video sources

Video sources categorized were academic (research group or colleges/universities), physician (independent or physician groups without research or college/university ties), non-physician (healthcare providers that are not physicians), athletic trainers, medical sources (content or animations from health websites), or commercial.

Video content

Content categories included the following: exercise training (Achilles tendon therapy and rehabilitation), disease-specific information, patient experiences/anecdotes, surgical approaches and/or techniques, non-surgical intervention or therapy, or advertisements.

Video reliability and educational content assessment

The Journal of the American Medical Association (JAMA) benchmark criteria were utilized to assess the reliability and accuracy of each video. The JAMA benchmark criteria are four nonspecific and objective criteria that may be evaluated in online resources. For each of the four criteria (Table 1) present in each video, a point is given for a maximum score of four and a minimum score of zero. A higher score is indicative of greater reliability and accuracy [15]. JAMA benchmark criteria have not been validated but have been used extensively to evaluate the reliability of online resources in previously published studies [6].

**Table 1:** Journal of the American Medical Association benchmark criteria Adapted from [15].

Criteria	Description
Authorship	Author and contributor credentials and their affiliations should be provided.
Attribution	Clearly lists all copyright information and states references and sources for content.
Currency	Initial date of posted content and subsequent updates to content should be provided.
Disclosure	Conflicts of interest, funding, sponsorship, advertising, support, and video ownership should be fully disclosed.

Assessment of nonspecific educational content quality was done via the Global Quality Score (GQS). GQS is a non-validated but commonly used metric used to assess the quality of online resources [6]. GQS utilizes five criteria to evaluate the educational content of online resources. For each of the five criteria (Table 2) present in each video, a point is given with a maximum score of five and a minimum score of zero, with a higher score being indicative of the greater quality of educational content [16].

**Table 2:** Global Quality Score criteria Adapted from [16].

Grading	Description of quality
1	Poor quality: is unlikely to be useful for patient education.
2	Poor quality: is of limited use to patients because only some information is present.
3	Suboptimal quality and flow: is somewhat useful to patients; important topics are missing; some information is present.
4	Good quality and flow: useful to patients because most important topics are covered.
5	Excellent quality and flow: is highly useful to patients.

Assessment of Achilles tendon-specific educational content was done via the Achilles Tendon Specific Score (ATSS). ATSS is composed of 19 criteria derived from the American Academy of Orthopaedic Surgeons (AAOS) guidelines and adapted from similar metrics in prior studies [6,17,18]. For each of the 19 criteria (Table 3) present in each video, a point is given with a maximum score of 19 and a minimum score of zero. A higher score is indicative of more comprehensive coverage of Achilles tendon function and pathologies. The use of novel unvalidated subject-specific scores to evaluate online content’s educational quality has been utilized in prior studies on orthopedic-related topics [6]. All criteria in the ATSS were categorized into patient presentation, information about the Achilles tendon, diagnosis and evaluation of Achilles tendon pathologies, treatment of Achilles tendon pathologies, and postoperative timeline, course, and outcomes.

**Table 3:** Achilles Tendon Specific Score and specific educational content Adapted from [6,17,18].

Achilles Tendon Specific Score categories
Patient presentation
Symptoms
Population and risk factors
Information about Achilles tendon
Anatomy/function
Regenerative ability
Acute vs. chronic tears & injuries
Partial vs. complete tears
Diagnosis and evaluation
Physical exam
X-ray ability
MRI vs. ultrasound gold standard
Surgical candidates
Surgical non-candidates
Treatment
Conservative treatment & physical therapy
Non-operative timeline
Open repair
Percutaneous repair
Post-operative course
Complications and outcomes
Physical/weight-bearing restrictions
Physical therapy
Timeline

Statistical analysis

All statistical analyses were performed in SPSS version 27.1 (IBM Corp., Armonk, NY). Descriptive statistics such as means, standard deviations, minimums, maximums, frequencies, and percentages were used to quantify characteristics, reliability, and quality scores (JAMA benchmark criteria, GQS, and ATSS) for each video. Each video was independently rated by four authors for JAMA benchmark criteria, GQS scores, and ATSS scores. None of the authors were aware of the scores given by other authors as all authors recorded their evaluations on separate spreadsheets. After all four authors had evaluated all 50 videos for JAMA benchmark criteria, GQS scores, and ATSS scores, data were collated in SPSS and analyzed for mean scores. Intra-observer reliability scores for JAMA benchmark criteria, GQS scores, and ATSS scores were then calculated using reliability analysis.

Distributive tests for the normalcy of data were performed with Kolmogorov-Smirnov and Shapiro-Wilk tests. One-way analysis of variance (ANOVA) (normally distributed data) and Kruskal-Wallis (non-normally distributed data) tests were used to determine whether video reliability and quality varied by video sources and content.

Multivariate linear stepwise regression was performed to evaluate the effects of specific video characteristics, content type, or video source on JAMA benchmark criteria score (reliability), GQS (non-specific educational quality), and ATTS (specific educational quality). P-values < 0.05 were considered statistically significant.

## Results

The first 50 YouTube videos obtained from the search were evaluated as none of them met exclusion criteria. These videos had a cumulative view total of 53,323,307, and the other video characteristics are listed in Table 4.

**Table 4:** YouTube video characteristics View ratio = views ÷ days since upload. Like ratio = (likes * 100) ÷ (likes + dislikes). Video power index (VPI) = like ratio * view ratio ÷ 100.

Video characteristic	Mean	Median	Standard deviation	Minimum	Maximum
Video duration (minutes)	4.97	4.26	4.18	0.88	23.68
Views	1,066,466	54,528	6,025,850	1,009	42,663,665
Likes	15,328	506	95,840	9	679,000
Dislikes	605	14.5	3,812	0	27,000
Comments	197	35.55	653	0	4,525
Days since upload	1,475	1,366	1,064	131	3,950
View ratio	6,647	58	46,039	1.06	325,677
Like ratio	95.55	96.8	4.27	76.6	100
Video power index (VPI)	6,392	55.3	44,279	1.06	313,222

**Figure 1:**
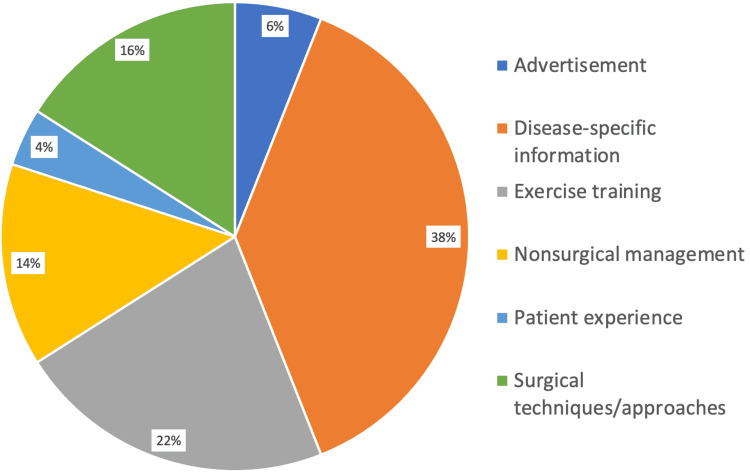
Relative frequencies of video content for Achilles-related YouTube videos

**Figure 2:**
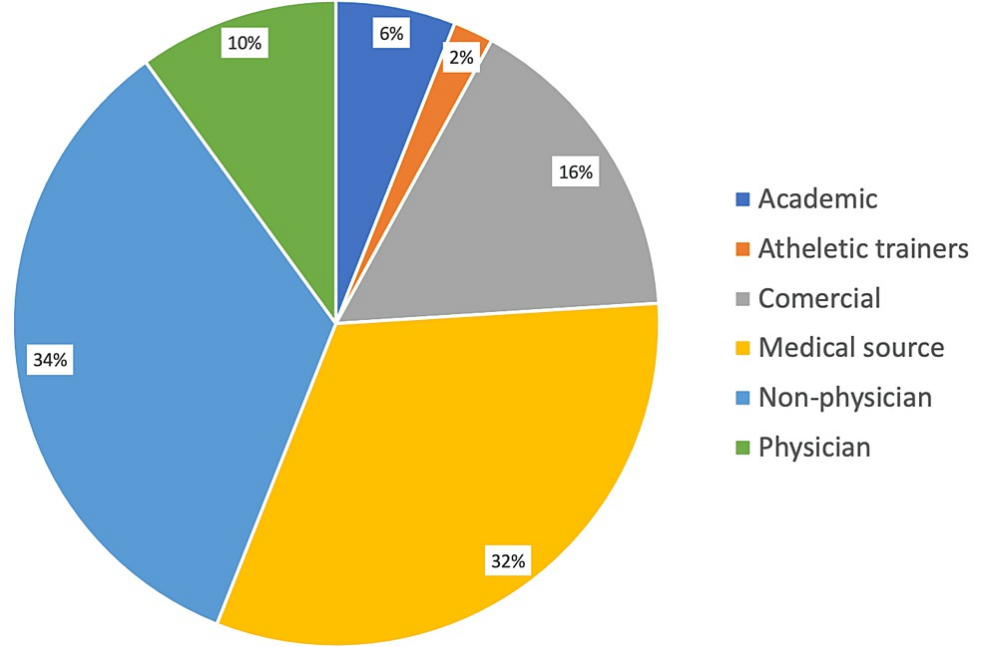
Relative frequencies of video upload source for Achilles-related YouTube videos

The most common information presented in videos was disease-specific information (38%) followed by exercise training (22%), surgical techniques (16%), and nonsurgical management (14%) (Figure 1). The most common source of videos was non-physicians (34%) (which includes physical therapists, physiotherapists, chiropractors, and podiatrists) and medical sources (32%) such as health websites/groups (Figure 2).

The mean JAMA benchmark criteria score was 2.69, GQS was 2.64, and ATSS was 4.66. Interobserver reliability was 0.405 (0.058-0.0644) for JAMA benchmark criteria, 0.851 (0.764-0.910) for GQS, and 0.925 (0.860-0.959) for ATSS.

Analysis of variance (Table 5) showed no statistically significant relationship between content type and JAMA benchmark criteria (P: 0.169), GQS (P: 0.857), ATSS (P: 0.331), or VPI (P: 0.445) scores or between upload source and JAMA benchmark criteria (P: 0.110), GQS (P: 0.523), ATSS (P: 0.526), or VPI (P: 0.568) scores (P > 0.05).

**Table 5:** Mean JAMA benchmark criteria, GQS, and ATSS scores for video content types and sources Higher scores indicate better reliability and accuracy, nonspecific educational content, or specific educational content. † No means as there was only one video uploaded by athletic trainers. JAMA: Journal of the American Medical Association; GQS: Global Quality Score; ATSS: Achilles Tendon Specific Score.

Variable group	JAMA mean (SD)	GQS mean (SD)	ATSS mean (SD)
Video content			
Exercise training	2.7 (0.6)	2.6 (0.4)	3.1 (1.7)
Disease-specific	2.8 (0.4)	2.7 (1.0)	5.4 (3.3)
Patient experience	2.1 (0.2)	1.8 (0.4)	3.6 (0.2)
Surgical treatment	2.5 (0.4)	2.5 (1.3)	5.0 (3.6)
Non-surgical	2.9 (0.2)	2.8 (0.9)	5.3 (2.3)
Advertisement	2.4 (0.6)	2.8 (0.9)	3.8 (3.8)
Video source			
Academic	2.8 (0.1)	2.2 (0.6)	3.2 (0.9)
Physician	3.0 (0.3)	2.9 (1.2)	6.2 (3.6)
Non-physician	2.8 (0.5)	2.8 (0.6)	4.4 (2.3)
Athletic trainers	2.3^†^	2.5^†^	2.5^†^
Medical sources	2.4 (0.5)	2.7 (1.1)	5.3 (3.4)
Commercials	2.6 (0.4)	2.3 (1.1)	3.8 (3.3)

Multivariate stepwise linear regression analysis was run to assess if there were independent associations between video characteristics, content, or source with JAMA benchmark criteria, GQS, or ATSS scores. Analysis showed that videos with increased view ratios (more views per day) had lower overall JAMA benchmark criteria scores (standardized beta = −0.281, P = 0.048). Videos with longer duration had increased GQS scores (standardized beta = 0.380, P = 0.007) and ATSS scores (standardized beta = 0.364, P = 0.009). No independent associations were found between uploader source or content and JAMA benchmark criteria, GQS, or ATSS scores.

## Discussion

The first 50 videos queued in this study had a very large number of views with a cumulative total of 53,323,307, a mean of 1,066,466, and a range of 1,009 to 42,663,665 views per video. These results are like previous studies on the quality of YouTube-derived content related to orthopedic topics such as kyphosis, posterior cruciate ligament (PCL), or disc herniation [6,19]. In a similar YouTube study on the PCL, Kunze et al. (2019) reported a view total of 14,141,285 with a mean of 50,478 views [6]. These findings are significantly lower than this current study suggesting that Achilles tendon information has a larger viewership and that understanding this could lead to better cooperation between patients and physicians for a large group of viewers.

Overall, the authors of this study found the reliability and accuracy, non-specific educational content, and specific educational content of individual videos to be of low quality. The mean JAMA benchmark criteria score of 2.69 out of 4.0 suggests that the reliability and accuracy of individual YouTube video content are moderate to low on average. The mean GQS score of 2.64 out of 5.0 suggests that the nonspecific educational quality of individual YouTube video content is low and that the ability of any single video to sufficiently inform consumers is suboptimal to poor. The mean ATSS score of 4.66 out of 19 suggests that the specific educational content in individual videos is not comprehensive and that they give patients only a fraction of all the information they would need to have a good understanding of the Achilles tendon and its pathologies. These findings suggesting low quality were like studies such as Kunze et al. (2019), which reported a mean JAMA benchmark criteria score of 2.02 out of 4, GQS score of 2.3 out of 5, and posterior cruciate ligament score (PCLS) of 2.9 out of 18 [6].

Though individual YouTube videos do not provide viewers with comprehensive educational content on the Achilles tendon, a person searching for this information online is unlikely to only obtain information from one video or one source. Thus, if a patient were to view multiple videos, reference other sources (such as mayoclinic.org or orthoinfo.aaos.org), and then consult with their physician, they could form a loose basis for understanding their condition and be better able to convey their goals and expectations. Though the content of YouTube videos is lacking in reliability and overall content, it is not generally created to be used as a comprehensive guide. Instead, if implemented correctly, usage of these videos may act as a primer to help further educate patients and provide them with some sense of control in their treatment, plan, and care.

Notably, no statistically significant associations were found between video uploader source or video content and the JAMA benchmark criteria, GQS, or ATSS scores of Achilles tendon-related videos. This suggests that video quality and reliability are not related to the source or content type. However, this study found that a higher view ratio related to a lower JAMA benchmark criteria score indicates that videos with greater popularity are less explicit with authorship, attribution, currency, and/or disclosure of conflicts of interest. GQS and ATSS were only increased with increased video duration, which is reasonable as a longer video would allow the uploader more time to provide a better and more comprehensive understanding of that specific topic.

If the educational content of these videos is to be accurate and thus maximize its benefit to the patient, it should follow the peer-reviewed reliable principles and guidelines established by the AAOS, which were adapted into the ATSS of this study. As stated above, the content of individual YouTube videos is of low quality and non-comprehensive but could act as a primer to help patients better understand their condition. When used in conjunction with high-quality resources such as the AAOS website, these videos could help ease patients into a position where they are more comfortable with their condition and more able to accurately and effectively communicate with their attending provider.

Limitations

This study was conducted on and analysis was limited to the first 50 YouTube videos queried using the term “Achilles tendon.” This limits the generalizability of this study but may mirror the actual search patterns of many users as they rarely search beyond the first or first few pages of results when searching a topic [20]. This style of evaluation for these first 50 videos with their high viewership is likely a valid approximation of the video quality and content of all Achilles tendon videos and has been likewise performed in previous studies like those cited above. The use of unvalidated tools for reviewing the quality and reliability of online resources in this study is like the common use of these same tools in many other published studies. The inter-observer reliability of the GQS and ATSS scores was excellent but was fair/poor for the JAMA benchmark criteria. This suggests that the evaluation of educational content in this study is robust while the evaluation of reliability was variable.

## Conclusions

The internet is becoming an increasingly powerful tool to obtain many forms of information, and as people become more used to using this tool, it is important that healthcare providers be aware of its use and potential for misuse. There is largely no peer-reviewed or regulatory system in place for YouTube videos or many online sources. Thus, providers should engage with their patients by asking them if they have used online resources to understand their condition and should help patients better utilize these resources. YouTube is a massive and growing free platform with millions of users and videos that are easily accessible and often used even though the information provided there is of low quality and lacking in content. Other resources such as the AAOS or Mayo Clinic websites are of higher quality but are far less popular, less utilized, and/or mostly provide information as text and images, which is often less engaging and more intimidating than the dynamic nature of videos where the source is walking you through a topic. The internet and the use thereof will only continue to grow in the coming years. If providers are able to properly address these resources with patients, participate in resource creation, and inform patients about their use, they may be able to help patients find a sense of control in their care. Subsequently, patients may find grounds to better discuss their knowledge with their provider instead of simply being educated by them. Through this cooperation, patients can seek out proper care at the appropriate time, thus reducing the risk of exacerbating injuries and future tendon ruptures.
